# Prevalence of gingivitis and calculus in 12-year-old Puerto Ricans: a cross-sectional study

**DOI:** 10.1186/s12903-017-0471-5

**Published:** 2018-01-19

**Authors:** Augusto R. Elias-Boneta, Karol Ramirez, Sona Rivas-Tumanyan, Margarita Murillo, Milagros J. Toro

**Affiliations:** 1 School of Dental Medicine, Medical Sciences Campus, University of Puerto Rico, Rio Piedras, Puerto Rico; 2 Faculty of Dentistry, University of Costa Rica, San Pedro Montes de Oca, San José, Costa Rica; 3 Neuroscience Research Center, University of Costa Rica, San Pedro Montes de Oca, San José, Costa Rica

**Keywords:** Gingivitis, Dental calculus, Bleeding on probing, Children, Puerto Rico, Prevalence

## Abstract

**Background:**

Gingivitis is a common oral health problem. Untreated gingivitis may progress to periodontitis, a common cause of tooth loss. The prevalence of gingivitis and calculus among Puerto Rican children is unknown. Understanding this prevalence can support early public health preventative strategies. This study aims to estimate the prevalence of gingivitis and calculus among 12-year-old Puerto Ricans by health region and to explore differences in distribution by school type (proxy for socio-economic status) and gender.

**Methods:**

A probability-based sample of 113 schools was selected proportional to enrollment size and stratified by health region, school type, and gender. Two trained examiners evaluated the presence of gingivitis and both supragingival and subgingival dental calculus. Gingivitis was defined as the presence of gingival bleeding upon gentle probing (BOP) in at least one site, and the extent of the problem was classified according to the percentage of teeth whose gingiva presented BOP (limited: 25–49% of the teeth tested; extensive: >50% of teeth tested). Logistic and linear regression models, adjusted for health regions, were used to compare gingivitis and calculus prevalence and extent between genders and school types.

**Results:**

Gingivitis was found in 80.41% of the 1586 children evaluated. Urban-public schoolchildren had a slightly higher prevalence (83.24%) compared to private (79.15%, *p* = 0.16); those in rural-public (77.59%) and private schools had similar prevalence (*p* = 0.15). Extensive gingivitis was present in 60.81% of all children. The mean percentage of sites presenting BOP (BOP%) was 17.79%. Rural and urban public schoolchildren presented significantly higher BOP% compared to children from private schools (*p* = 0.0005, *p* = 0.002, respectively). Dental calculus was detected in 61.59% of the sample, boys presenting significantly higher (*p* = 0.005) total and supragingival calculus. Rural-public schoolchildren had a significantly higher prevalence of subgingival calculus compared to private schoolchildren (*p* = 0.02).

**Conclusions:**

Gingivitis prevalence is higher among 12-year-old Puerto Ricans compared to data reported for U.S. adolescents. Public schoolchildren presented significantly higher BOP% sites compared to private schoolchildren. Boys presented a significantly higher total and supragingival calculus prevalence than girls. Oral health disparities related to gender and school type were identified by this study. Studies exploring the reasons for these disparities are recommended.

**Electronic supplementary material:**

The online version of this article (10.1186/s12903-017-0471-5) contains supplementary material, which is available to authorized users.

## Background

Gingivitis, defined as the presence of gingival bleeding in at least one site [1], is a mild form of periodontal disease and a common oral health problem [2]. Plaque-induced gingivitis is the most common type of gingivitis [3]. Gingival inflammation is exacerbated during puberty due to the expression of intra-cellular steroid hormone receptors in human gingival cells [4] and to an increase in steroid hormone levels [5]. Serum levels of testosterone in boys and estradiol and progesterone in girls are positively associated with *Prevotella (P.) intermedia* and *P. nigrescens* levels [6]. The relationship between elevated levels of circulating sex hormones and prevalence of gingivitis in puberty is evidenced by an earlier gingivitis peak in girls (11–13 years) than in boys (13–14 years) [6]. During puberty, periodontal tissues may have an amplified response to dental plaque, calculus, food debris, and materia alba [7]. Other risk factors for gingivitis include poor oral hygiene [8], high sugar consumption [8], and social determinants of health, such as economic inequalities [9].

Dental calculus, an important gingivitis-contributing factor, is a mineralized dental plaque deposit that forms on dental surfaces above (supragingival) and/or below (subgingival) the gingival margin. Dental calculus provides a substratum for plaque retention in the vicinity to the gingiva [10]. Supragingival calculus plays a minor role in the progression of periodontal disease [11]. However, subgingival calculus, along with gingival inflammation, is a determinant of disease progression in early-onset periodontitis [12]. Plaque-induced gingivitis initiates pocket formation and increases the flow of mineral-rich gingival fluid favoring the formation of subgingival calculus [10].

The worldwide prevalence of gingivitis and calculus in 12-year-olds varies widely from 23% to 100% [13–17]. A U.S. survey conducted by the National Institute of Dental Research (NIDR) during 1986–87 revealed that nearly 60% of children aged 14–17 had gingivitis, one-third presented supragingival calculus, and almost one-fourth exhibited subgingival calculus [18].

Gingivitis is an inducible and reversible disease. However, untreated gingivitis typically progresses to periodontitis, a more severe condition [19]. Therefore, gingivitis management is a prevention strategy for advanced periodontal disease [20]. Gingivitis is a common problem in adult Puerto Ricans [21]. Moreover, the prevalence of periodontitis in this population is relatively high affecting 45% of 70–79-year-olds [22], nearly twice the prevalence found in adults 65 and older in the U.S. [23]. In addition, several longitudinal clinical studies on both initiation and progression of periodontitis (mostly aggressive forms) in adolescents and young adults have shown that calculus, both subgingival and supragingival, is a risk factor for disease initiation [24] and strongly associated with disease prevalence [25]. These studies implicate calculus in the development of periodontitis and other periodontal conditions including gingival recession [12].

Little is known about the prevalence of gingivitis and calculus among schoolchildren in Puerto Rico. Such information is valuable in evaluating the oral health needs of Puerto Ricans and to plan strategies for gingivitis management, and the prevention of more advanced periodontal disease. The aim of this population-based, cross-sectional study is to estimate the prevalence of gingivitis and calculus among 12-year-olds residing in Puerto Rico during the academic year 2010–2011. It will then explore any differences in distribution between gender and school type (public and private). School type was used as a proxy for socio-economic status (SES).

## Methods

### Study design

This research is part of a comprehensive cross-sectional study that assessed the oral health status of 12-year-olds attending public and private schools in the eleven government health insurance regions (GHI) of Puerto Rico, using a multi-stage sampling methodology. The study design and sampling methodology have been previously described [26].

### Setting and sample selection

This study was conducted in schools, and the GHI regions were selected, as in 1997, to allow comparison with other oral health indicators assessed in a previous study [27]. The universe of public and private, urban and rural, schools was used as the sampling frame for this island-wide study. Schools were stratified according to their GHI region and, within each stratum, schools were organized according to their geographic proximity and poverty level. School level clustering reasonably assumed a social-cultural homogeneity at the school level. In Puerto Rico public/private school attendance is considered a proxy measure for SES, with private school attendance representing a higher family SES compared to public school attendance [26–29].

### Recruitment

Study recruitment occurred during October–November 2010 and January–April 2011. The study’s clinical procedures were conducted between November 2010–May 2011. After obtaining permission from the Department of Education and approval from the Institutional Review Board of the Medical Sciences Campus, University of Puerto Rico (MSC-UPR), the project director visited the selected schools in the sample. During each visit, the project director explained the study aims to the school principal and home-room teachers, and requested a list of 12-year-olds enrolled in 5th to 7th grades. Twenty 12-year-olds, 10 boys and 10 girls, were then randomly selected using a computer-generated random number. The project director and the home-room teacher explained the aims and methods of the study to the selected children, emphasizing that their participation was voluntary. A letter of invitation, informed consent form, medical history questionnaire and a demographic information form were sent to the parents of potential participants. The medical history questionnaire asked for details of any medical conditions suffered by the child, medications being taken and food allergies. Demographic information collected included the date of birth, gender, ethnicity, dental insurance information and home address.

### Inclusion and exclusion criteria

Inclusion criteria were as follows: 1) classification with a physical status ASA I and/or ASA II as defined by the American Society of Anaesthesiologist [30]; 2) being 12 years of age at the time of recruitment. The exclusion criteria were: 1) participants with conditions requiring antibiotic prophylaxis; 2) those who demonstrated an inability to comply with study protocol requirements.

### Study procedures

Prior to the study, the two study examiners were trained by a periodontist and an experienced reference examiner in gingivitis and calculus assessment. During the study, 10% of the examinations were repeated, and the examiners demonstrated 83.33% inter-examiner agreement on BOP scores and 88.89% agreement for calculus.

**Fig. 1:**
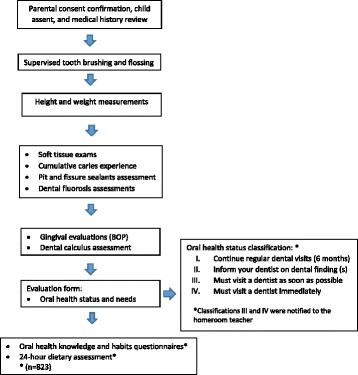
Study visit procedure

All clinical procedures were conducted in a single visit to the child’s school, after confirming proper parental and child consent, in the following sequence: 1. medical history review; 2. height and weight measurements; 3. soft tissue examinations; 4. cumulative caries experience (DMFS) and pit and fissure sealants; 5. dental fluorosis assessment; 6. gingival evaluations (BOP); 7. dental calculus assessment. As part of the main study, a sub-sample of the participants (*n* = 823) completed an oral health knowledge and habits questionnaire [see Additional file 1] and a 24-h dietary assessment [29]. A study procedure flowchart is included (Fig. 1).

The day the clinical exams were scheduled, qualifying children were asked to brush and floss their teeth under the home-room teacher’s supervision. As a significant number of children in public schools participate in the school breakfast and lunch programs, these oral hygiene procedures were useful in removing food debris and allowed better visualization of the oral hard and soft tissues.

The examiners conducted oral examinations and evaluated the presence of gingival bleeding and dental calculus using a modified version of the Oral Health Surveys of the National Institute of Dental Research Diagnostic Criteria and Procedures [31]. The portable equipment used consisted of a dental chair, external light source, and an air compressor. All processes complied with Occupational Safety and Health Administration (OSHA) infection control procedures. Oral examinations were conducted on all the erupted teeth in two selected quadrants (one maxillary and one mandibular) that were chosen using a computer-generated random number. Gingival and calculus status were not assessed around teeth with extensive dentinal caries, sub-optimal restorations or orthodontic bands/brackets since these areas are prone to plaque stagnation and inappropriate oral hygiene [32], and may produce overestimates of gingivitis prevalence and severity. Gingivitis was defined as the presence of BOP in at least one site [1]. The gingival sulcus was explored using a Hu-Friedy PCP UNC 126 periodontal probe. The probe was inserted not more than 2 mm into the gingival sulcus starting just distal to the midpoint of the buccal surface and then gently moved into the mesial interproximal area. The same procedure was completed on the palatal surface. Bleeding sites were scored after the sites of a single quadrant were probed. Each site was scored as no bleeding =0 and bleeding =1. Gingivitis extent was determined for each participant as follows: (a) limited gingivitis: 2–4 teeth or 25% to 49% of the teeth examined presented gingival bleeding; (b) extensive gingivitis: >5 teeth or >50% of the teeth examined presented gingival bleeding [33]. Children with gingivitis who did not fulfill these criteria were regarded as having localized gingival inflammation [34]. When assessing the presence of dental calculus, supragingival calculus was defined as calcified deposits located on the exposed crown and root surfaces extending up to 1 mm below the free gingival margin (FGM) and subgingival calculus was defined as mineralized deposits located more than 1 mm below the FGM. Supragingival calculus was assessed visually while using compressed air to dry teeth. A gentle tactile exam used the probe to locate subgingival calculus deposits. Each site was scored as follows: 0 = no calculus; 1 = supragingival calculus in the absence of subgingival calculus; 2 = presence of subgingival calculus only or presence of both supragingival and subgingival calculus; Y = site cannot be assessed (missing, partially erupted or deciduous tooth) [35]. The mid-buccal (buccal), mesio-buccal (buccal/mesial), mid-palatal (palatal), and mesio-palatal (palatal/mesial) surfaces were evaluated. Data were recorded on a modified NIDCR data entry form [35]. Dental plaque, probing depth, and clinical attachment level were not assessed. No radiographic examinations were performed.

After completing the oral examination, all children received an oral evaluation form to take home. This form classified the child’s oral health status from I-IV (Fig. 1) according to the severity of the oral findings and recommended the timing of their next dental visit. Those children with subgingival calculus were classified as III with the recommendation being that they “must visit a dentist as soon as possible”.

### Study size

The main objective of the study, which provided the source data for this analysis, was to estimate the prevalence and distribution of caries in this population. Consequently, sample size calculations were based on the expected DMFS score values, as described earlier [26]. In order to achieve the required final sample size of 1500 students, 133 schools (102 public and 31 private) were selected across the 11 regions, assuming 75% eligibility and 75% response rate, with 2660 students being invited to participate (2660*0.75*0.75 = 1496) [26].

### Statistical methods

Only three of the 32 private schools selected in the sample were located in rural areas. Therefore, all the private schools were grouped into a single category. Since this sample was stratified by GHI regions and clustered within schools, the analysis used weights inversely proportional to the probability of selection, adjusted for non-response, and later normalized. Estimated weighted percentages (95% confidence intervals, CI) were calculated for categorical measures (gingivitis and calculus prevalence, gingivitis and calculus extent), and percent of sites bleeding on probing (BOP%) was summarized in terms of weighted means (95% CI) and standard errors around the means. Differences in gender and school type, depending on the outcome measure being assessed, employed logistic, polytomous logistic and linear regression models, while taking into account the stratified cluster sample design and adjusting for health regions. Gingivitis prevalence (yes/no) and calculus prevalence (yes/no) were studied using logistic regression; gingivitis extent (extensive, limited, none) and calculus extent (supragingival, subgingival, none) were regressed using polytomous models; percent of sites with BOP (continuous) were regressed using linear models. All models included gender, school type (private, rural-public, urban-public) and health regions. Data were recorded in Microsoft Excel, verified and imported into the SAS statistical software package version 9.3 (SAS Statistical Institute, Cary, NC) for analyses.

## Results

**Fig. 2:**
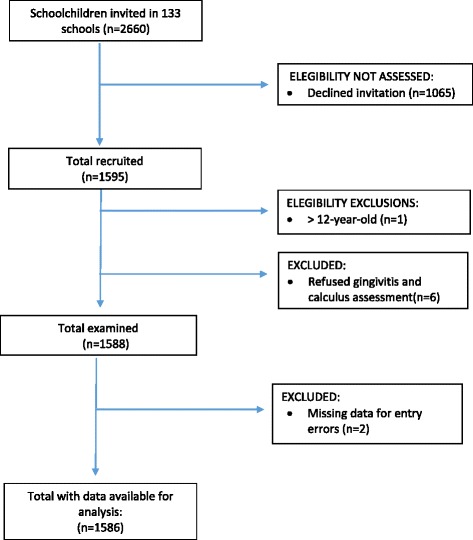
Participation and exclusion

A total of 1586 children participated in the study. The composition of this sample was 51% female, 49% male, with 84.05% from public schools, and 15.95% from private schools. Figure 2 presents a flow diagram of study participation rates and reasons for exclusion from the study. For each child between 7 and 14 teeth were evaluated (mean: 13.31), with 4 sites being assessed per tooth. The total number of sites evaluated per child ranged between 26 and 56 (mean: 53.16). On average, 0.68 teeth (4.9% of teeth) per child were not evaluated since they were missing (not yet erupted, or extracted) or excludable (dental caries, extensive restorations or orthodontic bands or brackets).

### Gingivitis

Gingivitis was identified in 80.41% (95% CI: 76.60, 84.21) of the schoolchildren. Geographically, prevalence across the 11 GHI regions (see Table 1) ranged from 72.36% in the East to 92.61% in the Northwest. Table 2 presents gingivitis prevalence and extent by gender and school type. Although gingival bleeding was more common in boys (81.29%) than girls (79.57%), this was not statistically significant (school type-and region-adjusted *p* = 0.54). The prevalence of gingivitis was highest in children attending urban-public schools (83.25%) by comparison to private (79.15%) or rural-public (77.59%) schools, but these differences were also not statistically significant after adjusting for gender and region (*p* = 0.16 for urban-public and *p* = 0.15 for rural-public). In terms of the extent of gingivitis, 19.59% (95% CI: 16.77, 22.45) of children had limited gingivitis, and 60.81% (95% CI: 56.03, 65.59) had extensive gingivitis. No statistically significant differences in gingivitis extent were observed between schoolchildren by gender (*p* = 0.64) or school type (rural-public *p* = 0.09 and urban-public *p* = 0.07).

**Table 1:** Weighted estimates for gingivitis prevalence in 12-year-old Puerto Ricans by region^a^, 2011

					Gingivitis		Limited Gingivitis		Extensive Gingivitis		BOP% Sites^c^		
Region	*N*	% N	Wt^b^ N	Wt N%	%	95% CI	%	95% CI	%	95% CI	Mean	SE	95% CI
North	165	10.40	297	18.73	72.68	(60.73–84.64)	20.30	(12.47–28.14)	52.38	(38.43–66.32)	14.77	1.351	(11.89–17.65)
East	129	8.13	69	4.35	72.36	(56.75–87.97)	31.69	(22.31–41.08)	40.67	(26.14–55.19)	12.01	1.466	(8.69–15.33)
Metropolitan	363	22.89	581	36.63	89.16	(84.04–94.28)	15.60	(10.55–20.66)	73.56	(66.41–80.70)	21.20	1.186	(18.77–23.62)
San Juan	163	10.28	164	10.34	76.75	(66.13–87.37)	21.07	(9.55–32.59)	55.68	(42.75–68.61)	18.69	3.093	(12.06–25.33)
Central	222	14.00	243	15.32	73.26	(61.33–85.20)	19.12	(12.89–25.36)	54.14	(41.20–67.08)	16.17	2.245	(11.41–20.93)
Southeast	143	9.02	67	4.22	74.07	(67.44–80.70)	24.74	(13.94–35.53)	49.34	(39.53–59.14)	14.21	1.234	(11.42–17.00)
Ponce	68	4.29	29	1.83	77.86	(58.11–97.60)	22.35	(15.94–28.75)	55.51	(34.90–76.12)	14.15	2.526	(7.66–20.64)
Southwest	82	5.17	13	0.82	74.86	(67.13–82.59)	26.67	(17.02–36.32)	48.19	(31.84–64.54)	14.46	2.690	(6.99–21.93)
West	78	4.92	47	2.96	80.40	(67.90–92.90)	23.32	(13.47–33.18)	57.07	(38.74–75.41)	17.00	1.987	(12.42–21.58)
Northwest	77	4.85	46	2.90	92.61	(82.67–100.00)	29.06	(7.47–50.65)	63.55	(42.36–84.74)	16.75	1.605	(12.95–20.54)
Northeast	96	6.05	30	1.89	83.87	(61.80–100.00)	20.08	(12.60–27.56)	63.79	(36.94–90.65)	18.97	3.215	(11.10–26.83)
Total PR	1,586	100	1,586	100	80.41	(76.60–84.21)	19.59	(16.77–22.42)	60.81	(56.03–65.59)	17.79	0.755	(16.30–19.29)

^a^Puerto Rico health administrative regions, as in 1997

^b^weighted

^c^percentage of bleeding on probing sites

**Table 2:** Gingivitis prevalence and extent in 12-year-old Puerto Ricans by gender and type of school, 2011

			Gingivitis			Limited Gingivitis			Extensive Gingivitis			BOP% Sites			
Strata	*N*	Wt N	%	95% CI	*p*-value^1a^	%	95% CI	*p*-value^2b^	%	95% CI	*p*-value^2^	Mean	SE	95% CI	*p*-value^3c^
Gender															
Female	841	814	79.57	(74.16–84.98)	0.54	18.75	(14.30–23.20)	0.48	60.82	(54.43–67.21)	0.64	17.69	0.932	(15.85–19.54)	0.83
Male	745	773	81.29	(77.40–85.17)		20.48	(16.58–24.39)		60.80	(55.21–66.39)		17.90	0.852	(16.21–19.58)	
School type															
Rural-public	597	612	77.59	(70.92–84.26)	0.15	21.66	(17.36–25.97)	0.67	55.92	(47.81–64.03)	0.09	16.82	1.168	(14.47–19.17)	<0.0005
Urban-public	628	722	83.24	(78.07–88.40)	0.16	16.36	(12.09–20.62)	0.96	66.88	(60.40–73.36)	0.07	19.44	1.191	(17.05–21.83)	<0.002
Private	361	253	79.15	(69.64–88.66)	–	23.82	(17.08–30.56)	–	55.33	(44.75–65.91)	–	15.44	1.223	(12.94–17.94)	–

^1a^*p*-values were obtained from multivariate logistic regression models, which included gender, school type (3 categories), and health region (11 regions)

^2b^*p*-values were obtained from multivariate polytomous logistic regression models (outcome categories: extensive, limited, no gingivitis), which included gender, school type (3 categories), and health region (11 regions)

^3c^*p*-values were obtained from multivariate linear regression models, which included gender, school type (3 categories), and health region (11 regions)

### Bleeding on probing (BOP) parameters

The mean BOP for each child was 9.46 sites, which accounted for 17.79% (95% CI: 16.30, 19.29) of the sites probed. The mean percentage of teeth presenting BOP was 43.54% (95% CI: 40.31, 46.77) or 5.80 of the actual teeth examined. Table 2 shows that children attending urban-public and rural-public schools presented significantly higher BOP% sites compared to children attending private schools (gender and region-adjusted, urban-public *p* = 0.002 and for rural-public vs. private *p* = 0.0005).

### Dental calculus

Dental calculus was identified in 61.59% (95% CI: 56.61, 66.57) of children (Table 3), with 57.09% having supragingival and 19.78% having subgingival calculus. As shown in Table 4, boys presented a significantly higher prevalence of total calculus (66.28%, 95% CI: 60.95, 71.60; *p* = 0.005) and supragingival calculus (62.06%, 95% CI: 56.59, 67.65; *p* = 0.001) than girls (57.31%, 95% CI: 51.10, 63.51 and 52.37%, 95% CI: 46.27, 58.47, respectively). Children attending rural-public schools had a significantly higher prevalence of subgingival calculus (20.8%, 95% CI: 15.42, 26.18) compared to children in private schools (15.98%, 95% CI: 7.94–24.01; gender and region adjusted *p* = 0.02).

**Table 3:** Weighted estimates for calculus prevalence and extent in 12-year-old Puerto Ricans by region^a^, 2011

					Total Calculus		Supragingival Calculus		Subgingival Calculus^c^	
Region	*N*	% N	Wt^b^ N	Wt N%	%	95% CI	%	95% CI	%	95% CI
North	165	10.40	297	18.73	45.42	(32.59–58.25)	36.09	(25.43–46.75)	18.36	(7.97–28.75)
East	129	8.13	69	4.35	61.56	(45.78–77.33)	55.82	(41.76–69.87)	15.02	(5.02–25.03)
Metropolitan	363	22.89	581	36.63	69.41	(60.31–78.51)	66.96	(58.08–75.84)	20.30	(11.85–28.73)
San Juan	163	10.28	164	10.34	75.79	(62.80–88.78)	73.84	(61.33–86.34)	27.04	(15.34–38.74)
Central	222	14.00	243	15.32	53.78	(44.31–63.25)	49.45	(41.01–57.89)	15.84	(10.79–20.89)
Southeast	143	9.02	67	4.22	64.67	(54.73–74.61)	60.79	(49.82–71.76)	16.15	(9.63–22.68)
Ponce	68	4.29	29	1.83	43.04	(31.73–54.35)	41.31	(31.28–51.33)	8.29	(0.00–17.06)
Southwest	82	5.17	13	0.82	74.39	(64.70–84.08)	63.05	(53.04–73.06)	27.18	(16.17–38.19)
West	78	4.92	47	2.96	64.35	(49.19–79.50)	57.96	(42.81–73.10)	25.50	(11.41–39.60)
Northwest	77	4.85	46	2.90	51.55	(26.80–76.30)	42.93	(14.37–71.49)	24.87	(10.65–39.08)
Northeast	96	6.05	30	1.89	72.40	(59.79–85.01)	71.43	(59.59–83.29)	26.22	(15.01–37.43)
Total PR	1,586	100	1,586	100	61.59	(56.61–66.57)	57.09	(52.03–62.15)	19.78	(16.01–23.55)

^a^Puerto Rico government health insurance regions, as in 1997

^b^weighted

^c^alone or accompanied by supragingival calculus

**Table 4:** Calculus prevalence and extent in 12-year-old Puerto Ricans by gender and school, 2011

			Total Calculus (yes/no)			Supragingival Calculus			Subgingival Calculus^a^		
Strata	N	Wt N	%	95% CI	*p*-value^2b^	%	95% CI	*p*-value^2b^	%	95% CI	*p*-value^2b^
Gender											
Female	841	814	57.31	(51.10–63.51)	0.005	52.37	(46.27–58.47)	0.001	18.34	(13.35–23.32)	0.34
Male	745	773	66.28	(60.95–71.60)		62.06	(56.59–67.65)		21.30	(16.72–25.88)	
School Type											
Rural- public	597	612	52.68	(45.93–59.44)	0.63	46.07	(39.13–53.01)	0.47	20.80	(15.42–26.18)	0.02
Urban- public	628	722	67.39	(59.49–75.29)	0.55	64.18	(56.57–71.80)	0.47	20.25	(13.73–26.76)	0.23
Private	361	253	66.60	(57.23–75.98)	–	63.49	(55.08–71.89)	–	15.98	(7.94–24.01)	–

^a^alone or accompanied by supragingival calculus

^2b^*p*-values were obtained from multivariate logistic regression models (outcome categories: supragingival, subgingival calculus, and none), which included gender, school type (3 categories), and health region (11 regions)

## Discussion

This is the first island-wide representative sample study to assess the prevalence of gingivitis and dental calculus in Puerto Rican children. The study found a high prevalence of both gingivitis and calculus in its subjects. Gingival bleeding in children attending urban-public schools was marginally higher than in private and rural-public school. Children attending rural-public schools had a lower prevalence of dental calculus compared to children attending private and public-urban schools, but a significantly higher prevalence of subgingival calculus compared to children in private schools. Boys presented a significantly higher total calculus and supragingival calculus compared to girls.

In terms of limitations, our population only included 12-year-old children. However, this is the suggested standard assessment age for international comparisons [35]. Due to the age of our population sample, and the study setting, we decided not to collect dental plaque data. This is because the most appropriate method for dental plaque detection involves the use of a disclosing solution [36], and, in a school setting, this would prove cumbersome, complicating study flow and extending the procedure duration. By excluding children with severe systemic conditions or special health care needs, who constitute a group prone to gingival inflammation and sub-optimal oral health, we may have underestimated the overall prevalence and severity of plaque-induced gingivitis. Similarly, since we did not record probing depth nor clinical attachment level during the clinical assessment, children presenting subgingival calculus may have suffered more advanced periodontal disease. Partial-mouth examinations, in this case, two quadrants per child, may have resulted in an underestimation of gingivitis and calculus prevalence [37]. However, to minimize this limitation the examined quadrants were randomly assigned and only a small number of teeth, less than 5%, were necessarily excluded. By contrast, the pre-exam oral hygiene performance might have increased rates of BOP [38].

The prevalence of gingivitis in children varies globally. Studies in China and Yemen report gingivitis in most children [17, 39] while studies in Mexico report prevalence lower [40] than reported here. These variations may be due to cultural differences in oral habits or reflect subtle differences in study methodology. The only gingivitis data available for U.S. schoolchildren arises from the National Survey of Oral Health, conducted by the National Institute of Dental Research (NIDR) during 1986–87 [18]. This reported that nearly 60% of 14–17-year-olds presented with gingivitis, with more than half of children in both genders having extensive gingivitis [18]. However, these data are not readily comparable with our findings since they are sourced from older adolescents and the study is now outdated.

Our study found a higher prevalence of dental calculus than that reported in other studies, including both the U.S. survey of children aged 14–17 years old [18] and a study of 12-year-olds in China [17]. This finding is repeated in other studies in the international literature [1, 41, 42].

In terms of SES variations, an oral health study of 12-year-old Brazilians revealed a higher rate of BOP in children attending public schools [15]. This finding concurs with our study, which found that public school attendees, rural and urban, had significantly higher rates of BOP by comparison to private school attendees. This BOP variation could arise from differences in oral hygiene, dietary habits, education, and health promotion activities. These associations will be explored in our future publications using this data set. In our study, children attending public schools had higher rates of subgingival calculus. Two previous studies from Brazil also found that calculus prevalence was lower in 12-year-olds attending private school compared to those attending public schools [43, 44]. Lower rates of calculus have been reported among children attending private school compared to those attending public school [45].

With regard to variations between urban and rural communities, a study based in the country of Georgia found a significantly higher prevalence of bleeding on probing in adolescents from urban communities compared to rural areas [45]. By contrast, a study in North Jordan found that rural adolescents and adults presented a higher prevalence of gingivitis and periodontitis than their urban peers [1]. In our study, urban public school children had higher rates of BOP% than their rural public school peers.

Most epidemiological studies show that gingivitis is more prevalent in adolescent [46, 47] and adult [33, 48] males. However, studies of 12-year-olds have shown, as has ours, no significant difference in gingivitis prevalence between the genders [17]. This may be due to age-related hormonal changes occurring at an earlier age in girls [6], thereby exacerbating their pubertal gingivitis. Less appropriate oral hygiene habits in boys likely explain the gender difference in calculus formation that our study observed. Multiple environmental and systemic factors effect periodontal health, including oral hygiene habits, oral health knowledge, and SES [1]. For example, in older teens, a reduction in dental plaque and decline in gingivitis has been reported [49], and this may be related to improved oral hygiene in response to social pressures.

Gingivitis is a reversible disease. Prevention and early treatment would improve the level of gingival health and may prevent the development and progression of periodontal disease and its potential systemic complications [50]. Oral hygiene behaviors acquired during pre-adolescence often continue throughout life [51], and so early oral health education is a valuable public health strategy in the prevention and control of gingivitis.

The results of this study served as a basis for the establishment of a school-based primary preventive program in the municipality of Coamo, Puerto Rico. To improve the oral health of these children the program includes, among other measures, oral health promotion, supervised teeth brushing, and referral for comprehensive oral evaluation and prophylactic care.

With this in mind, this research highlights the need for further study of the causative factors underlying oral health disparities in Puerto Rican children. Additional longitudinal studies, encompassing the entire island, and drawing on a larger sample size, should also include assessments of dental plaque, pocket depth, and loss of attachment assessment to evaluate periodontal health trends. Such ongoing studies would support the design and delivery of appropriate preventive and therapeutic public health strategies.

## Conclusion

Puerto Rican children suffer from high rates of gingivitis and dental calculus, with nearly 60% of the island-wide sample presenting with extensive gingivitis and/or calculus. Oral health disparities related to school type and gender were identified. Public schoolchildren presented a significantly higher rate of BOP sites compared to private schoolchildren and, given that school type was used here as a socioeconomic proxy, this is an indication of ongoing economic disparities. Boys presented a significantly higher total and supragingival calculus prevalence. These results indicate the need for early preventative strategies to be designed and implemented to help preempt the development of more advanced periodontal disease. These findings both justify and assist in the design of, carefully planned and targeted strategies of prevention.

## Additional file

Additional file 1: English version of the Oral Health Knowledge and Habits Questionnaire. 26 item Oral Health Knowledge and Habits Questionnaire. (DOCX 20 kb)

## Abbreviations

ASA: American Society of Anesthesiologist
BOP: Bleeding on probing
DMFS: Decayed missing filled surface
FGM: Free gingival margin
GHI: Government health insurance
IL-1: Interleukin 1
MSC: Medical sciences campus
NIDR: National Institute of Dental Research
PR: Puerto Rico
SES: Socio-economic status
TNF: Tumor necrosis factor
U.S.: United States
UPR: University of Puerto Rico

## References

[1] Ababneh KT, Abu Hwaij ZMF, Khader YS (2012). Prevalence and risk indicators of gingivitis and periodontitis in a multi-centre study in North Jordan: a cross sectional study. BMC Oral Health..

[2] Albandar JM (2002). Global risk factors and risk indicators for periodontal diseases. Periodontol.

[3] Parameter on plaque-induced gingivitis. American Academy of Periodontology. J Periodontol. 2000;71 Suppl 5:851–2. 10.1902/jop.2000.71.5-S.851 10875689

[4] Güncü GN, Tözüm TF, Cağlayan F (2005). Effects of endogenous sex hormones on the periodontium--review of literature. Aust Dent J.

[5] Al-Ghutaimel H, Riba H, Al-Kahtani S, Al-Duhaimi S (2014). Common periodontal diseases of children and adolescents. Int J Dent.

[6] Nakagawa S, Fujii H, Machida Y, Okuda K (1994). A longitudinal study from prepuberty to puberty of gingivitis. Correlation between the occurrence of Prevotella intermedia and sex hormones. J Clin Periodontol.

[7] Chaitra TR, Manuja N, Sinha AA, Kulkarni AU. Hormonal effect on gingiva: pubertal gingivitis. BMJ Case Rep. 2012; 10.1136/bcr.2012.006193. 10.1136/bcr.2012.006193 PMC3433508 22927275

[8] Jaghasi I, Hatahet W, Dashash M (2012). Dietary patterns and oral health in schoolchildren from Damascus, Syrian Arab Republic. East Mediterr Health J.

[9] Sheiham A, Nicolau B (2005). Evaluation of social and psychological factors in periodontal disease. Periodontol.

[10] Jepsen S, Deschner J, Braun A, Schwarz F, Eberhard J (2011). Calculus removal and the prevention of its formation. Periodontol.

[11] White DJ (1997). Dental calculus: recent insights into occurrence, formation, prevention, removal and oral health effects of supragingival and subgingival deposits. Eur J Oral Sci.

[12] Albandar JM, Kingman A, Brown LJ, Löe H (1998). Gingival inflammation and subgingival calculus as determinants of disease progression in early-onset periodontitis. J Clin Periodontol.

[13] Botero JE, Rösing CK, Duque A, Jaramillo A, Contreras A (2015). Periodontal disease in children and adolescents of Latin America. Periodontol.

[14] Zhang S, Liu J, Lo ECM, Chu C-H (2014). Dental and periodontal status of 12-year-old Bulang children in China. BMC Oral Health..

[15] Maltz M (2001). Barbachan e Silva B. [relationship among caries, gingivitis and fluorosis and socioeconomic status of school children]. Rev Saude Publica.

[16] Szpringer-Nodzak M, Moszczeńska-Cieślikowska B, Remiszewski A, Gieorgijewska J (1989). Assessment of the condition of the parodontium in children aged 12 years using the parodontal treatment needs index. Czas Stomatol.

[17] Zhang S, Xu B, Liu J, Lo EC, Chu C-H (2015). Dental and periodontal status of 12-year-old Dai school children in Yunnan Province, China: a cross-sectional study. BMC Oral Health..

[18] Bhat M (1991). Periodontal health of 14-17-year-old U.S. schoolchildren. J Public Health Dent.

[19] Robinson PJ (1995). Gingivitis: a prelude to periodontitis?. J Clin Dent.

[20] Chapple ILC, Van der Weijden F, Doerfer C, Herrera D, Shapira L, Polak D (2015). Primary prevention of periodontitis: managing gingivitis. J Clin Periodontol.

[21] Elías-Boneta AR, Encarnación A, Rivas-Tumanyan S, Berríos-Ouslán BC , García-Godoy B , Murillo M, Diaz-Nicolas J, et al. Prevalence of Gingivitis in a Group of 35- To 70-Year-Olds Residing in Puerto Rico P R Health Sci J. 2017;36:140-145. PMC12424442 28915302

[22] Montero-Aguilar M, Muñoz-Torres F, Elías-Boneta AR, Dye B, Joshipura KJ (2012). High levels of periodontal disease among the older adult population in San Juan, Puerto Rico. Community Dent Health.

[23] Dye BA, Tan S, Smith V, Lewis BG, Barker LK, Thornton-Evans G, Eke PI, Beltrán-Aguilar ED, Horowitz AM, Li CH (2007). Trends in oral health status: United States, 1988-1994 and 1999-2004. Vital Health Stat.

[24] Van der Velden U, Abbas F, Armand S, Loos B, Timmerman M (2006). Java project on periodontal diseases. The natural development of periodontitis: risk factors, risk predictors and risk determinants. J Clin Periodontol.

[25] Susin C, Haas AN, Valle PM, Oppermann RV, Albandar JM (2011). Prevalence and risk indicators for chronic periodontitis in adolescents and young adults in south Brazil. J Clin Periodontol.

[26] Elias-Boneta AR, Toro MJ, Rivas-Tumanyan S, Murillo M, Orraca L, Encarnacion A (2016). Persistent oral health disparity in 12-year-old Hispanics: a cross-sectional study. BMC Oral Health.

[27] Elías-Boneta AR, Crespo Kebler K, Gierbolini CC, Toro Vizcarrondo CE, Psoter WJ (2003). Dental caries prevalence of twelve year olds in Puerto Rico. Community Dent Health.

[28] Morales OE (2012). La importancia de la preparación universitaria en estudiantes en desventaja social y económica. Revista Griot.

[29] Torres R, Santos E, Orraca L, Elias A, Palacios C (2014). Diet quality, social determinants, and weight status in puerto rican children aged 12 years. J Acad Nutr Diet.

[30] Daabiss M (2011). American Society of Anaesthesiologists physical status classification. Indian J Anaesth.

[31] National Institute of Dental Research (1991). Epidemiology and oral disease prevention program. Diagnostic criteria and procedures. Oral health surveys of the National Institute of dental research.

[32] Pari A, Ilango P, Subbareddy V, Katamreddy V, Parthasarthy H (2014). Gingival diseases in childhood - a review. J Clin Diagn Res.

[33] Albandar JM, Kingman A (1999). Gingival recession, gingival bleeding, and dental calculus in adults 30 years of age and older in the United States, 1988-1994. J Periodontol.

[34] Lang N, Adler R, Joss A, Nyman S (1990). Absence of bleeding on probing. An indicator of periodontal stability. J Clin Periodontol.

[35] World Health Organization (1997). Oral health surveys: basic methods.

[36] Wilkins EM, Wilkins EM, Wyche CJ (2013). Patient learning for health behavioral change. Clinical practice of the dental hygienist.

[37] Albandar JM (2011). Underestimation of Periodontitis in NHANES surveys. J Periodontol.

[38] Abbas F, Voss S, Nijboer A, Hart AA, Van der Velden U (1990). The effect of mechanical oral hygiene procedures on bleeding on probing. J Clin Periodontol.

[39] Al-Haddad KA, Al-Hebshi NN, Al-Ak’hali MS (2010). Oral health status and treatment needs among school children in Sana’a City, Yemen. Int J Dent Hyg.

[40] Ortega-Maldonado M, Mota-Sanhua V, López-Vivanco JC (2007). Oral health status of adolescents in México City. Rev Salud Publica (Bogota).

[41] Agbelusi GA, Jeboda SO (2006). Oral health status of 12-year-old Nigerian children. West Afr J Med.

[42] Mittal M, Chaudhary P, Chopra R, Khattar V (2014). Oral health status of 5 years and 12 years old school going children in rural Gurgaon, India: an epidemiological study. J Indian Soc Pedod Prev Dent.

[43] Nogueira dos Santos NC, TDB A, Freitas VS, Jamelli SR, Cavalcanti Sarinho ES (2007). Oral health among adolescents: aspects relating to hygiene, dental cavities and periodontal disease in the cities of Recife and Feira de Santana, Brazil. Cien Saude Colet.

[44] Freire Mdo CM, Reis SCGB, Gonçalves MM, Balbo PL, Leles CR (2010). Oral health in 12 year-old students from public and private schools in the city of Goiânia, Brazil. Rev Panam Salud Publica.

[45] Levin L, Margvelashvili V, Bilder L, Kalandadze M, Tsintsadze N, Machtei EE (2013). Periodontal status among adolescents in Georgia. A pathfinder study. PeerJ.

[46] Taani DQ (2001). Trends in oral hygiene, gingival status and dental caries experience in 13-14-year-old Jordanian school children between 1993 and 1999. Int Dent J.

[47] Ericsson JS, Abrahamsson KH, Ostberg A-L, Hellström M-K, Jönsson K, Wennström JL (2009). Periodontal health status in Swedish adolescents: an epidemiological, cross-sectional study. Swed Dent J.

[48] Australian Research Centre for Population Oral Health, The University of Adelaide SA (2009). Periodontal diseases in the Australian adult population. Aust Dent J.

[49] Gökalp SG, Doğan BG, Tekçiçek MT, Berberoğlu A, Unlüer S (2010). National survey of oral health status of children and adults in Turkey. Community Dent Health.

[50] Shangase SL, Mohangi GU, Hassam-Essa S, Wood NH (2013). The association between periodontitis and systemic health: an overview. SADJ.

[51] Saied-Moallemi Z, Virtanen JI, Vehkalahti MM, Tehranchi A, Murtomaa H (2009). School-based intervention to promote preadolescents’ gingival health: a community trial. Community Dent Oral Epidemiol.

