# Supplementary material for: Prevalence of gingivitis and calculus in 12-year-old Puerto Ricans: a cross-sectional study
Source: BMC Oral Health. 2018 Jan 19;18:13. doi: 10.1186/s12903-017-0471-5 (PMC5775617; doi:10.1186/s12903-017-0471-5)
Supplement: Additional file 1: — English version of the Oral Health Knowledge and Habits Questionnaire. 26 item Oral Health Knowledge and Habits Questionnaire. (DOCX 20 kb) [file 12903_2017_471_MOESM1_ESM.docx]

Date of birth: day ______ month______ year ________ Age: ____________

Interviewer initials: _______________

Start time of the interview: ______________

Student gender: F ___ M ___

Interviewer: "We're going to ask you some questions about your knowledge and oral health habits. We would be grateful if you reply as sincerely as possible”

1. Have you been taught how to brush your teeth?

___ (1) Yes

___ (2) No

___ (88) I do not know

___ (99) Refused

2. Where did they teach you to brush your teeth? (Check all that apply with [X], read all the premises)

___ (1) At home

___ (2) At school

___ (3) At the dentist's office

___ (4) Watching television

___ (5) In other place (s) Where: _________________

___ (6) Nobody

___ (88) I do not know

___ (99) Refused

3. What is dental plaque?

___ (1) A tooth decay or a broken tooth

___ (2) A gum disease

___ (3) A layer of bacteria and food stuck to the teeth

___ (88) I do not know

___ (99) Refused

4. What is dental caries?

___ (1) A broken tooth caused by bacteria and food that accumulates in the teeth

___ (2) A dirty tooth

___ (3) A tooth that has food

___ (4) Same as dental plaque

___ (88) I do not know

___ (99) Refused

5. What is periodontal disease or gum disease?

___ (1) A disease that affects the support of the teeth

___ (2) A disease of the tongue

___ (3) When you get a black tooth

___ (88) I do not know

___ (99) Refused

6. Do you know what oral cancer is?

___ (1) A broken tooth caused by bacteria and food that accumulates in the teeth

___ (2) A disease that affects the gums and the support of the teeth

___ (3) A disease involving abnormal and malignant growth in the mouth or tongue

___ (88) I do not know

___ (99) Refused

7. What is dental floss used for?

___ (1) To clean tooth decay

___ (2) To clean dental plaque between teeth

___ (3) To clean the part of the tooth that is used to chew

___ (88) I do not know

___ (99) Refused

8. What is fluoride used for?

___ (1) To clean the teeth

___ (2) To eliminate tooth decay

___ (3) To make teeth more resistant and prevent cavities

___ (88) I do not know

___ (99) Refused

9. Is it important for you to take care of your teeth?

___ (1) Yes

___ (2) No

___ (88) I do not know

___ (99) Refused

10. Do you think your teeth are healthy?

___ (1) Yes

___ (2) No

___ (88) I do not know

___ (99) Refused

11. Do you think your gums are healthy?

___ (1) Yes

___ (2) No

___ (88) I do not know

___ (99) Refused

12. Do you think that the appearance of your teeth (how they look) limit you to participating in activities at school or sharing with other friends?

___ (1) Yes

___ (2) No

___ (88) I do not know

___ (99) Refused

13. Do you think the condition of your mouth can affect your overall health?

___ (1) Yes

___ (2) No

___ (88) I do not know

___ (99) Refused

14. Would you like to have more guidance on oral health and hygiene in your school?

___ (1) Yes

___ (2) No

___ (88) I do not know

___ (99) Refused

15. Have you been taught to use a mouthwash? (Interviewer give examples)

___ (1) Yes

___ (2) No

___ (88) I do not know

___ (99) Refused

16. Where did you learn how to use a mouthwash? (Check all that apply with [X], read all the premises)

___ (1) At home house

___ (2) At school

___ (3) At the dentist's office

___ (4) Watching television

___ (5) In other place (s) Where? ___________________

___ (6) No one showed me

___ (88) I do not know

___ (99) Refused

17. Have you been taught to floss?

___ (1) Yes

___ (2) No

___ (88) I do not know

___ (99) Refused

18. Where were you taught to floss? (Check all that apply with [X], read all the premises)

___ (1) At home

___ (2) At school

___ (3) At the dentist's office

___ (4) Watching television

___ (5) In other place (s) Where? ____________________

___ (6) No one showed me

___ (88) I do not know

___ (99) Refused

19. What do you think people brush their teeth for? (Check all that apply with [X], read all the premises)

___ (1) So that I do not have a bad breath

___ (2) So that the teeth are not damaged

___ (3) To remove plaque

___ (4) So that I do not have to go to the dentist

___ (5) To keep teeth clean

___ (6) So that my gums do not bleed any more

___ (7) To feel a fresh mouth

___ (88) I do not know

___ (99) Refused

Dentist Visits

Interviewer: "I'm going to ask you some questions about your oral health status."

20. Have you ever visited a dentist?

___ (1) Yes 🡪 2

___ (2) No 🡪 4

___ (88) I do not know

___ (99) Refused

21. How many times in the past year (in the past 12 months) have you visited a dentist? (Read all the premises)

___ (1) once a year

___ (2) 2 times a year

___ (3) Other frequency: __________

___ (4) I did not go to the dentist last year (in the past 12 months)

___ (88) I do not know

___ (99) Refused

22. Why do you visit a dentist? (Check all that apply with [X], read all the premises)

___ (1) For a checkup

___ (2) When my teeth hurt

___ (3) When teeth need cleaning

___ (4) To get my teeth straight

___ (5) When I think I have a damaged tooth

___ (6) For other problem (s) (e.g. gum problems) Specify: ______________________

___ (7) I do not go to the dentist

___ (88) I do not know

___ (99) Refused

23. Is it difficult for you to visit a dentist?

___ (1) Yes Please explain why: _____________________________________________

___ (2) No

___ (88) I do not know

___ (99) Refuse

24. How many times a day do you brush your teeth?

___ (1) I never brush my teeth

___ (2) I do not brush my teeth every day

___ (3) Once a day

___ (4) Twice a day

___ (5) Three times a day

___ (6) More than three times a day

___ (88) I do not know

___ (99) Refused

25. How often do you floss? (Check all that apply with [X], read all the premises)

___ (1) Once a day

___ (2) Several times a day

___ (3) I do not floss

___ (4) Flossing before bedtime

___ (5) Flossing after each meal

___ (88) I do not know

___ (99) Refused

26. How often do you use mouthwashes? (Check all that apply with [X], read all the premises)

___ (1) Once a day

___ (2) Several times a day

___ (3) I do not use a mouthwash

___ (4) I use the mouthwash before bedtime

___ (5) I use a mouthwash after every meal

___ (88) I do not know

___ (99) Refused

End of interview

Time I Finish the Interview: _______________________

Interviewer: "This was my last question. Responses from all participants in this study will be combined and analyzed to develop preventive strategies to improve the oral health of children like you.

Thank you for your time and cooperation!
